# High-density genetic mapping of a major QTL for resistance to multiple races of loose smut in a tetraploid wheat cross

**DOI:** 10.1371/journal.pone.0192261

**Published:** 2018-02-27

**Authors:** Sachin Kumar, Ron E. Knox, Asheesh K. Singh, Ron M. DePauw, Heather L. Campbell, Julio Isidro-Sanchez, Fran R. Clarke, Curtis J. Pozniak, Amidou N’Daye, Brad Meyer, Andrew Sharpe, Yuefeng Ruan, Richard D. Cuthbert, Daryl Somers, George Fedak

**Affiliations:** 1 Department of Genetics and Plant Breeding, Chaudhary Charan Singh University, Meerut, Uttar Pradesh, India; 2 Swift Current Research and Development Centre, Agriculture and Agri-Food Canada, Swift Current, Saskatchewan, Canada; 3 1501 Agronomy Hall, Iowa State University, Ames, Iowa, United States of America; 4 Advancing Wheat Technology, Swift Current, Saskatchewan, Canada; 5 School of Agriculture and Food Science, University College Dublin, Belfield, Dublin, Ireland; 6 Department of Plant Sciences and Crop Development Centre, University of Saskatchewan, Saskatoon, Saskatchewan, Canada; 7 Global Institute of Food Security, University of Saskatchewan, Saskatoon, Saskatchewan, Canada; 8 Vineland Research and Innovation Centre, Vineland Station, Ontario, Canada; 9 Ottawa Research and Development Centre, Agriculture and Agri-Food Canada, Ottawa, Ontario, Canada; Institute of Genetics and Developmental Biology Chinese Academy of Sciences, CHINA

## Abstract

Loose smut, caused by *Ustilago tritici* (Pers.) Rostr., is a systemic disease of tetraploid durum wheat (*Triticum turgidum* L.). Loose smut can be economically controlled by growing resistant varieties, making it important to find and deploy new sources of resistance. Blackbird, a variety of *T*. *turgidum* L. subsp. *carthlicum* (Nevski) A. Love & D. Love, carries a high level of resistance to loose smut. Blackbird was crossed with the loose smut susceptible durum cultivar Strongfield to produce a doubled haploid (DH) mapping population. The parents and progenies were inoculated with *U*. *tritici* races T26, T32 and T33 individually and as a mixture at Swift Current, Canada in 2011 and 2012 and loose smut incidence (LSI) was assessed. Genotyping of the DH population and parents using an Infinium iSelect 90K single nucleotide polymorphism (SNP) array identified 12,952 polymorphic SNPs. The SNPs and 426 SSRs (previously genotyped in the same population) were mapped to 16 linkage groups spanning 3008.4 cM at an average inter-marker space of 0.2 cM in a high-density genetic map. Composite interval mapping analysis revealed three significant quantitative trait loci (QTL) for loose smut resistance on chromosomes 3A, 6B and 7A. The loose smut resistance QTL on 6B (*QUt*.*spa-6B*.*2*) and 7A (*QUt*.*spa-7A*.*2*) were derived from Blackbird. Strongfield contributed the minor QTL on 3A (*QUt*.*spa-3A*.*2*). The resistance on 6B was a stable major QTL effective against all individual races and the mixture of the three races; it explained up to 74% of the phenotypic variation. This study is the first attempt in durum wheat to identify and map loose smut resistance QTL using a high-density genetic map. The QTL *QUt*.*spa-6B*.*2* would be an effective source for breeding resistance to multiple races of the loose smut pathogen because it provides near-complete broad resistance to the predominant virulence on the Canadian prairies.

## Introduction

Durum wheat [*Triticum turgidum* L. subsp. durum (Desf.) Husn. (2n = 4× = 28 AABB genome)] is an important component of the diet of people in many regions of the world. Durum wheat is well adapted to semiarid climates and represents 5 to 8% of total wheat production [1, 2]. Canada ranks second after the European Union in durum wheat production with an average of 4.4 million tonnes each year (International Grain Council; http://www.igc.int/en/default.aspx). Most of the durum wheat cultivars registered for production on the Canadian prairies are susceptible to many races of the loose smut pathogen [3, 4].

Loose smut is caused by the fungus *Ustilago tritici* (Persoon) Rostrup, a seed-borne pathogen. It replaces the spike floral tissues with dark brown masses of teliospores, causing yield reduction approximately proportional to the percentage of smutted spikes. The infection process and life cycle of *U*. *tritici* on wheat have been studied and well documented [5]. In brief, the teliospores of *U*. *tritici* arrive in the floret and penetrate the ovary through germinating on the feathery stigma during anthesis [6]. At spore germination stage, if spores of more than one race of loose smut enter the floret at the same time, it is possible for sexual recombination to occur producing new virulence types within the pathogen. Mycelia of *U*. *tritici* survive within the embryo of infected seeds that do not differ from healthy seeds in appearance. Upon seed germination, the pathogen spreads systemically and penetrates through the growing point of the tillers without any apparent tissue damage; however, disease symptoms become visible on the spikes when they emerge from the boot. Numerous methods have been advocated for the control of loose smut infection in wheat but growing loose smut resistant varieties is one of the most effective approaches [7]. Although, loose smut resistant wheat varieties have been developed and grown, very little effort has been applied to the identification and mapping of genomic regions controlling infection of *U*. *tritici* races. Identification of favorable alleles and incorporation of loose smut resistance in high yielding, well-adapted wheat cultivars is desirable to reduce the need for chemical control measures and thereby reduce the environmental footprint.

Loose smut, a disease in which the pathogen demonstrates host specificity with the evolution of new pathogenic races over time, was extensively reviewed by [8, 9]. In an annual survey across Canada spanning 34 years (1964–1998), Menzies et al. [10] collected a total of 609 isolates of *U*. *tritici* that were examined for virulence on a set of differential tetraploid and hexaploid wheat cultivars. In the 1979–80 collection, two races of *U*. *tritici* T32 and T33 were identified from durum wheat for the first time. In subsequent survey years, as many as 241 isolates were assessed, from which 75% of isolates were detected as race T32 and 17% as T33. Another race, T26, was also identified that exhibited virulence to only one of the durum wheat differentials. Compared to T32 and T33, race T26 occurred at a low frequency in the pathogen population. Menzies et al. [10] proposed that the durum wheat differential hosts possessed different single genes (monogenic) for resistance to loose smut and virulence exists for all differentials. Durum cultivars are susceptible because of the wide virulence across races such as T26, T32 and T33 of *U*. *tritici*, which prompts the need to identify resistance to these races in durum. Knox et al. [11] reported on two qualitative genes for resistance in a durum wheat in which parents DT676 and W9260-BK03 of a doubled haploid (DH) mapping population each shared a single resistance gene to race T26 but differed for a second gene imparting resistance to races T32 and T33. Other quantitative oligo- or poly-genic inheritance for resistance against *U*. *tritici* in wheat is reported [9, 12], but single broad resistance genes are easier to select.

Breeding and studies of genetic inheritance of resistance to loose smut require phenotypic evaluation of segregating populations inoculated with *U*. *tritici* races. Phenotypic evaluation of loose smut expression requires two successive generations: the first generation for inoculation with *U*. *tritici* race(s), and second generation to determine disease infection response after heading. Phenotypic evaluation is optimized by artificial inoculation which is resource intensive and time-consuming. Even with careful inoculation escapes can occur which generate false-positive events that require additional testing to assure assignment of the correct phenotype.

Most investigations involving genetic analysis of loose smut resistance were conducted in hexaploid rather than durum wheat, which were summarized by Knox and Menzies [9]. Based on the information available, six genes, namely *Ut1*-*Ut6*, for loose smut resistance were identified and documented [13–15]. Knox et al. [16] mapped six quantitative trait loci (QTL) in hexaploid wheat on chromosomes 3A, 5B, 6B, 6D, 7A and 7B for resistance to single races and mixtures of multiple races of *U*. *tritici* that included T2, T9, T10, T15, T19, T27 and T39. They used simple sequence repeat (SSR) markers and four DH mapping populations. They related the QTL identified for loose smut resistance to the resistance genes reported earlier by Nielsen [17]. Krivchenko and Bakhareva [18] listed 52 genes for resistance to loose smut from 34 hexaploid wheat genotypes; 11 genes were reported recessive and the rest dominant. Some genotypes contained up to three resistance genes. Although it appears that a substantial number of resistance genes to loose smut exist, little is known about the expressivity, penetrance, and breadth of the resistance. Fewer resources and the need for smaller population size have historically favoured breeding programs deploying broad, major resistance genes into potential cultivars. With the large amount of work and cost required to perform traditional phenotypic testing of loose smut, interest has turned to the use of molecular markers for improved efficiency of selection.

A range of molecular markers such as restriction fragment length polymorphism (RFLP), amplified fragment length polymorphism (AFLP), SSR, diversity array technology (DArT) and single nucleotide polymorphism (SNP) were applied effectively to construct durum wheat genetic linkage maps [19–24]. A range of these marker types have been used at low-density to conduct QTL analysis for loose smut resistance including SSR, SCAR and AFLP markers [11, 16, 25, 26]. The resolution of the genetic maps is directly proportional to the marker density and size of a mapping population as markers mapped with high-density can localize recombination events more precisely. Therefore, increasing resolution can enhance the accuracy of QTL mapping and allow the discovery of new genomic regions affecting traits of interest.

In the present study, we performed high-throughput SNP genotyping using the Illumina Infinium II iSelect 90K SNP assay to construct a high-density genetic linkage map as a resource for SNP-based marker-trait association studies in durum wheat. The objective of the present study was to identify QTL for resistance to the loose smut pathogen *U*. *tritici* and characterize the effectiveness of the loci to multiple races.

## Materials and methods

### Genetic materials

The mapping population of 90 DH lines from a cross between two tetraploid wheat genotypes ‘Strongfield’ and ‘Blackbird’ was generated at Agriculture and Agri-Food Canada (AAFC) using the maize pollination technique [27]. The female parent Strongfield (*T*. *turgidum* L. var. *tetraploid*), a variety in the Canada Western Amber Durum wheat class [28], is moderately resistant to loose smut race T26, but highly susceptible to races T32 and T33. The male parent Blackbird [*T*. *turgidum* L. subsp. *carthlicum* (Nevski in Kom) A. Love & D. Love] accession REB68421 [27] caries a high level of resistance to *U*. *tritici* races T26, T32 and T33.

### Field experiment and phenotyping

The 90 DH lines, the parents ‘Strongfield’ and ‘Blackbird’ and susceptible check cultivars ‘Brigade’ [29], ‘Commander’ [30], ‘DT696’ and ‘AAC Raymore’ [31] were seeded in a randomized complete block design with three replications in field trials in 2011 and 2012 at Swift Current, Saskatchewan, Canada. A plot was one row, 3 m long with 200 seeds per row. Plots were 23 cm apart and separated by a row of spring-planted winter wheat that remained vegetative throughout the growing season to help control weeds and soil erosion, and to facilitate access for sampling. The experimental genotypes were examined for response to each of the *U*. *tritici* races T26, T32 and T33 as described by Knox et al. [11, 32]. These races were selected for evaluation because they represented prominent virulence across the Canadian prairies. Syringe and vacuum techniques of spore inoculation were used at mid-anthesis [33]. Inoculum (teliospore suspension) of an individual race was prepared at the rate of 1 g of teliospores per 1 L of tap water. In syringe inoculation, all florets on a spike were inoculated with the teliospore suspension of individual *U*. *tritici* races through a hypodermic needle. In each field trial, separate spikes in a plot were syringe-inoculated with individual races of T26, T32 and T33. In vacuum inoculation, the suspension of a mixture of all three races T26, T32 and T33 was used for inoculating at least two spikes [designated as head 1 (H1) and head 2 (H2)] of an experimental genotype in each plot across both years. Multiple spikes of the parents and check cultivars were inoculated with individual races and mixture of races. Inoculated spikes were labelled with a tag to indicate the particular race used. Inoculated spikes were harvested at harvest-maturity and separately hand threshed. The inoculated seeds were planted with no more than one spike per row in greenhouse beds for rating loose smut symptoms. The greenhouse temperature was set at 20 ± 5°C with a photoperiod of 16 h and 8 h dark. Each row was labeled to track head, genotype identity, and race treatment. The loose smut incidence (LSI) in each line for each individual race and mixture of races was determined as follows:

$$LSI\;(\%)=\frac{Number\;of\;smutted\;plants}{Total\;number\;of\;plants}\times 100$$

### DNA extraction, genotyping assay and data scoring

Fresh young leaves (3 to 6 cm in length) of the parents and 90 DH lines were excised for extracting and purifying high-quality genomic DNA using the BioSprint 96 workstation and the BioSprint 96 DNA Plant Kit (Qiagen Inc., Ontario, Canada) following the manufacturer’s instructions. Total DNA was then quantified using a Qubit^®^ 2.0 fluorometer (www.probes.invitrogen.com/qubit) to allow equalization of concentrations. Each sample was diluted to 250 ng/μl of DNA for SNP genotyping.

The parents and the population were genotyped with the Infinium II iSelect 90K SNP assay of wheat (referred to hereafter as wheat 90K assay) using the Illumina platform (Illumina Inc.) at the National Research Council-Plant Biotechnology Institute (NRC-PBI) and AAFC, Saskatoon, Canada. The wheat 90K assay contains a total of 81,587 functional (gene-associated) SNPs developed under the aegis of the International Wheat SNP Working Group [34, 35]. In brief, the Infinium II assay is based on one bead type per feature (or SNP) that employed whole-genome amplification through a single-base extension step for discriminating two alleles among experimental genotypes that is further detected with two fluorescence color assays by Illumina’s iScan reader [36]. The clustering and the genotype calling of each SNP was analysed using the genotyping module of GenomeStudio v2012 software (Illumina Inc.) with a GenCall score cut-off of 0.15 (as per Illumina’s recommendations). Clearly separated two or three clusters (according to SNP allele segregation in the parents and among the DH lines) were considered to be polymorphic SNPs. Several of the SNP clusters were compressed or overlapped and could not be separated by the default routine in GenomeStudio and were manually ascertained. Identified polymorphic SNPs were exported from GenomeStudio to a spreadsheet for subsequent data analysis.

### SNP genotyping

Genotyping with the wheat 90K assay generated data points for 81,587 functional SNPs. In the present study, these SNPs had attained an average call rate of 82% (range 0.79–0.85) for the Strongfield/Blackbird population. Numbers and positions of clusters for each SNP were taken into consideration during data visualization in GenomeStudio. Of 81,587 SNPs, 51,690 (63.4%) were monomorphic; 11,024 (13.5%) totally failed to give informative calls; and 5,921 (7.3%) produced indistinguishable clusters. Finally, 12,952 (15.8%) SNPs revealed polymorphism in the Strongfield/Blackbird population. Both alleles (i.e. A and B alleles) of polymorphic SNPs showed strong hybridization signals between Strongfield and Blackbird and hence were considered as co-dominant SNP markers. Different patterns of cluster distribution of SNPs from the 90 DH lines in two-dimensional analysis are presented in S1 Fig. A majority of polymorphic SNPs produced two main clusters indicating homozygous AA and homozygous BB alleles along the *theta*-axis with the standard cluster separation values (*theta*) 0.0 and 1.0. Nevertheless, a third cluster representing heterozygous AB alleles was also examined at *theta* 0.5, in which polymorphic SNPs appeared as two clusters representing homozygous AA and heterozygous AB alleles at *theta* 0.0 and 0.5, respectively. Similarly heterozygous AB and homozygous BB allele were also observed at *theta* 0.5 and 1.0, respectively. Dominant SNP markers exhibited no signal for one of the two alleles, revealing that only one allele was hybridized and another allele failed (null allele) to hybridize. A completely failed SNP with *R* < 0.10 where *R* represents intensity of hybridization signal occurred when there was no annealing with a target genomic region and hence produced a technically indistinct cluster (S1 Fig).

### Genetic map construction

Genotyping calls of 12,952 polymorphic SNPs on 90 DH lines along with the parents, obtained from GenomeStudio, were phased into allele scores in the spreadsheet. The SNPs were combined with genotyping data of 426 SSR markers (77 barc, 37 cfa, 23 cfd, 8 gdm, 100 gwm and 181 wmc) from the Strongfield/Blackbird DH population that was previously used to construct a SSR-based genetic map [27]. The complete dataset of 13,378 markers was employed for map construction using the software package MSTMap [37], which was further refined in MapDisto software [38] as described by Fowler et al. [39]. Markers with ≥10% missing data and incorrectly genotyped by not following parental alleles were removed from the dataset. The chi-square goodness-of-fit test (*p*<0.05) to the expected Mendelian segregation ratio of 1:1 was determined for each marker. Linkage groups were further checked individually to identify miscalls appearing as double crossovers which were imputed and analyzed. Recombination frequencies were translated to map distances in centimorgans (cM) using the Kosambi mapping function [40]. Assignment of SSR and SNP markers in linkage groups to wheat chromosomes was based on Somers et al. [41], Wang et al. [35], Maccaferri et al. [42]. The final chart of each linkage group was drawn using MapChart 2.2 software [43]. The high-density map contained many markers with a common segregation pattern (co-segregating markers) that mapped at the same position in genetic bins. Such markers offered no new information in the map and consumed much computation resources. Therefore, where more than one marker mapped in a genetic bin, the one marker with the least missing data was selected to represent that genetic bin and markers were remapped. Consequently, a map of 1,411 markers (SNPs + SSRs) was used for QTL discovery.

### Quantitative trait analysis and mapping

The mixed model analysis of variance was performed on each environment with PROC MIXED [44] implemented in Statistical Analysis Software (SAS) version 8.2 [45]. Genotypes were considered fixed and replications were random. Outliers in the data were diagnosed using the rstudent [46] option in SAS. The Least Square (LS) means function in the SAS program was used to calculate means of replications in each experiment for QTL analysis. A frequency distribution of LSI was plotted within the Strongfield/Blackbird DH population inoculated with individual races and mixture of races in trials from 2011 and 2012.

Quantitative trait locus (QTL) analysis of LSI was conducted for the Strongfield/Blackbird DH population using QTL Cartographer v2.5 software [47]. The composite interval mapping (CIM) function was used on the LS means data to determine the significant association of markers to the loose smut incidence. For background marker selection in CIM, the forward and backward regression of standard model 6 was used for QTL discovery. The walk speed used was 1 cM and the window size was set at 10 cM. A genome-wide threshold Logarithm of the odds (LOD) score for each trait was computed by the permutation test of 1,000 random iterations at a significance level of *P<0*.*05* [48] to declare significant QTL. The effect of identified QTL for a level of loose smut resistance was obtained as a percentage of phenotypic variation explained (% PVE).

## Results

### Phenotypic assessment of loose smut

**Fig 1:**
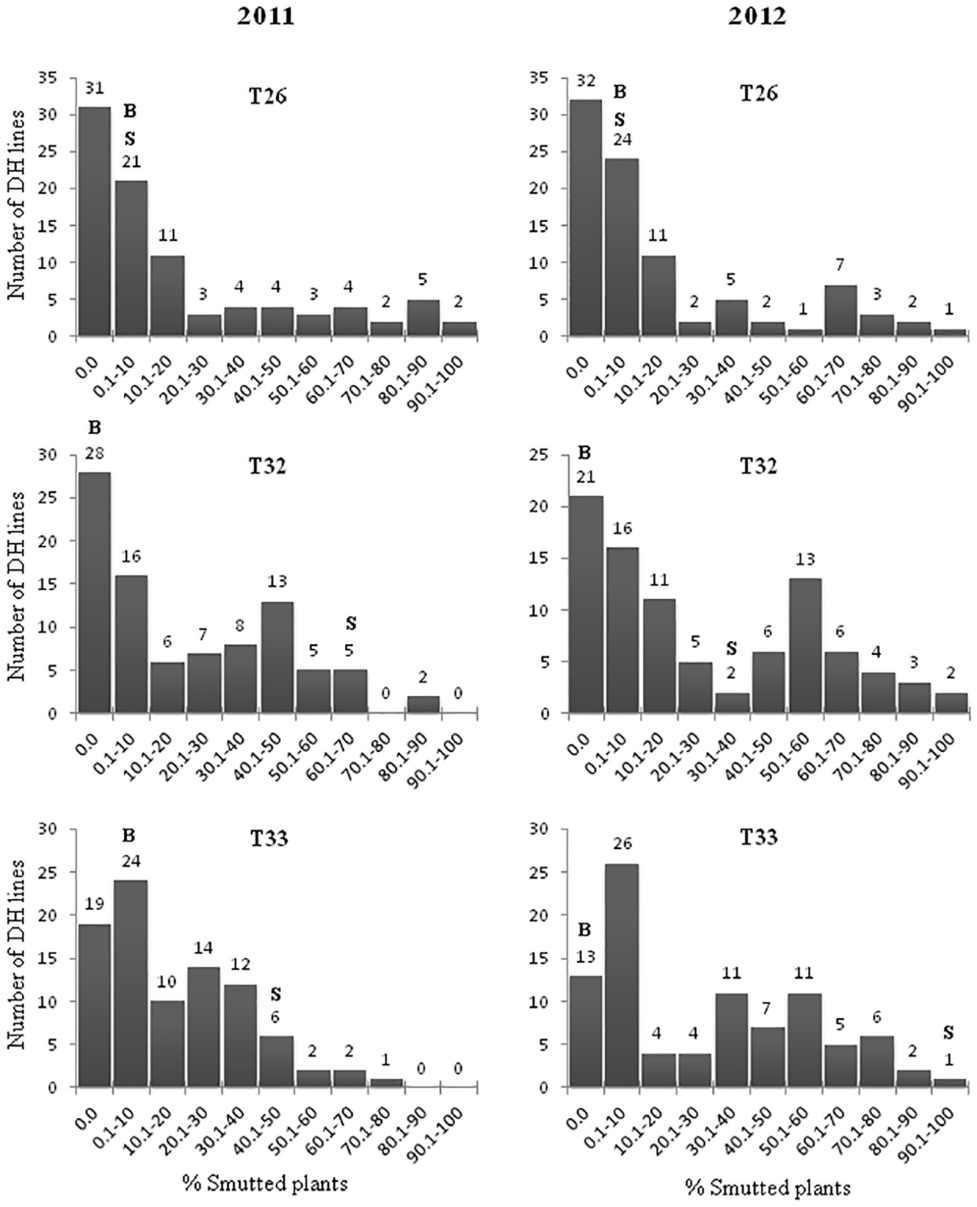
Phenotypic distribution of percent loose smut incidence (LSI, %) for the Strongfield/Blackbird DH mapping population in response to *Ustilago tritici* individual races T26, T32 and T33. Percent loose smut incidence levels for parents Blackbird and Strongfield are indicated by ‘B’ and ‘S’, respectively.

**Fig 2:**
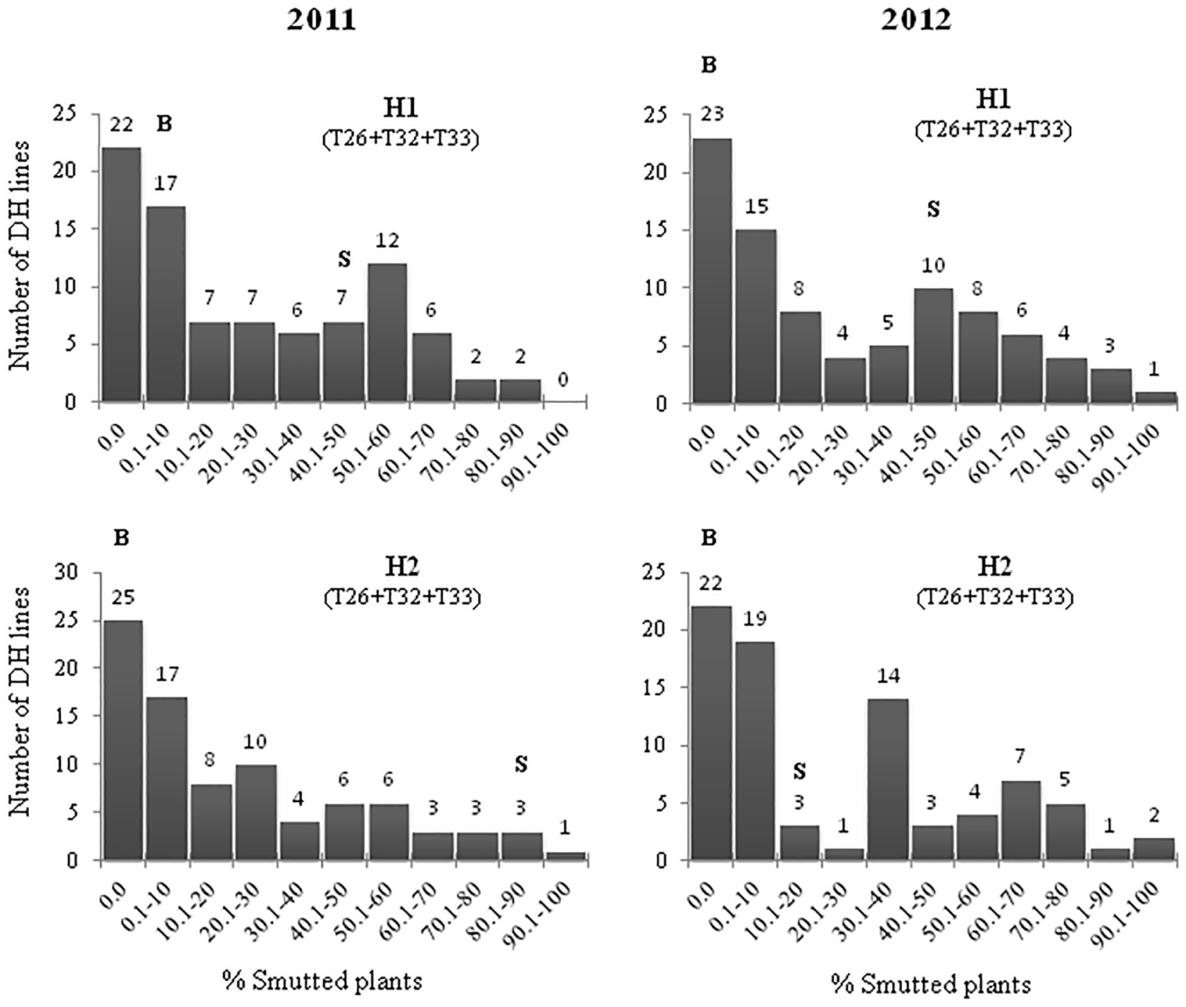
Phenotypic distribution of percent loose smut incidence (LSI, %) for the Strongfield/Blackbird DH mapping population in response to a mixture of *Ustilago tritici* races T26, T32 and T33. Percent loose smut incidence levels for parents ‘Blackbird’ and ‘Strongfield’ are indicated by ‘B’ and ‘S’, respectively. H1 represents head 1 and H2 represents head 2.

The susceptible check cultivars Brigade, Commander, DT696 and AAC Raymore were susceptible to each of the races T32 and T33, while they were moderately resistant to race T26 in both trials (2011 and 2012) (S1 Table). All the four check cultivars were susceptible when the mixture of three races (T26, T32 and T33) was used. The resistant parent Blackbird showed complete to nearly complete resistance to individual and mixed inoculation of races T26, T32 and T33 (Figs 1 and 2). Like the check cultivars, susceptible parent Strongfield was moderately resistant to race T26 but highly susceptible to individual races T32 and T33, while it was found to be susceptible to the mixture of races T26, T32 and T33 (S1 Table). A reduced level of disease infection was observed when the mixture of races was used for inoculation. For example, Commander exhibited a lower infection level to the mixture of races than races T32 and T33 used individually.

The frequency distributions of loose smut incidence (%) among Strongfield/Blackbird DH lines from trials in 2011 and 2012 are presented in Figs 1 and 2. Transgressive segregation was observed in both years with the distribution of loose smut incidence skewed such that trailing off in the susceptible tail occurred for race T26. It was difficult to clearly classify the population into resistant and susceptible classes, but a preponderance of resistant types was observed. Distributions appearing to have two peaks were observed for races T32 and T33 in both years.

### High-density genetic map

From the 13,378 SNP and SSR markers, 933 (918 SNPs + 15 SSRs) were excluded due to ≥10% missing data points and distorted segregation (p<0.05) from a ratio 1:1 over the 90 DH lines. The genetic map constructed with MSTMap and MapDisto software consisted of 16 linkage groups. This high-density genetic map comprised 12,445 (12,034 SNP + 411 SSRs) markers with a total genetic length of 3,008.4 cM and an average spacing of 0.2 cM between neighboring markers. The complete genetic map is presented in S2 Table and a summary of statistics is given in Table 1. The distribution of markers on wheat chromosomes is shown in S2 Fig. More markers were mapped on the B-genome (6968 spanned 1508.5 cM) than on the A-genome (5,477 spanned 1499.9 cM). Among the homoeologous groups, a maximum of 1994 markers was mapped on the group 2 chromosomes followed by 1937 on group 5 chromosomes and a minimum of 1446 on group 4 chromosomes. The linear order of mapped SSR and SNP markers in the present map resembled the marker order in genetic maps published by Somers et al. [41] for SSRs and Wang et al. [35], Maccaferi et al. [42] for SNPs. Genetic maps of chromosome 3B and 7A were each assembled in two linkage groups; labelled as 3B.1, 3B.2 and 7A.1, 7A.2 (Table 1; S2 Fig). The wheat SNPs and SSRs were well distributed throughout the genetic map.

**Table 1:** Summary of the total number of SNP and SSR markers distributed on the Strongfield/Blackbird genetic map.

Chromosome (parts)	No. of linkage group	Total number of mapped markers	No. of SNP markers	No. of SSR markers	Map length (cM)	Map resolution (cM)
1A	1	825	802	23	174.2	0.2
2A	1	679	648	31	225.9	0.3
3A	1	690	663	27	234.9	0.3
4A	1	725	693	32	231.3	0.3
5A	1	736	706	30	238.3	0.3
6A	1	919	904	15	192.3	0.2
7A (7A.1, 7A.2)	2	903	869	34	203.0	0.2
1B	1	846	816	30	199.9	0.2
2B	1	1,315	1,272	43	212.4	0.2
3B (3B.1, 3B.2)	2	976	940	36	233.5	0.2
4B	1	721	700	21	164.4	0.2
5B	1	1,201	1,171	30	275.7	0.2
6B	1	992	968	24	203.4	0.2
7B	1	917	882	35	219.2	0.2
A genome	8	5,477	5,285	192	1,499.9	0.3
B genome	8	6,968	6,749	219	1,508.5	0.2
AB genomes	16	12,445	12,034	411	3,008.4	0.2

### QTL analysis

The genome-wide significant LOD threshold for declaration of QTL was achieved by 1000 permutations (*P*<0.05) for each dataset, which ranged from 3.28 to 3.55. At these threshold levels, high-density SNP map-assisted composite interval mapping (CIM) analysis revealed three statistically significant QTL for loose smut resistance in the Strongfield/Blackbird population for syringe and vacuum inoculation treatments in 2011 and 2012. These QTL were located on chromosomes 3A, 6B and 7A. Designations of QTL, associated marker-intervals, LOD scores of QTL peaks, the proportion of phenotypic variation explained (%PVE), and favorable parental allele are presented in Table 2.

**Table 2:** Significant QTL identified for resistance to loose smut (*Ustilago tritici*) in the Strongfield/Blackbird DH population using composite interval mapping analysis.

*Ustilago tritici* race	Year	Chromosome name	QTL associated marker-interval^a^	Position (cM)	LOD threshold^b^	LOD score^c^	Percent PVE^d^	Additive value^e^	Favorable parental allele	QTL designation
T26	2011	3A	Kukri_c10751_264	150.5	3.3	9.4	19.8	12.9	Strongfield	*QUt*.*spa-3A*.*2*
T26	2012	3A	JG_c2645_107	151.9	3.3	9.9	21.2	12.1	Strongfield	*QUt*.*spa-3A*.*2*
T26	2011	6B	Tdurum_contig76997_244	110.7	3.3	16.1	39.2	-18.5	Blackbird	*QUt*.*spa-6B*.*2*
T26	2012	6B	Tdurum_contig76997_244	110.7	3.3	13.2	35.0	-15.5	Blackbird	*QUt*.*spa-6B*.*2*
T32	2011	6B	RAC875_c13216_111—Tdurum_contig76997_244	109.3	3.3	24.9	63.0	-19.2	Blackbird	*QUt*.*spa-6B*.*2*
T32	2012	6B	Tdurum_contig76997_244—barc24	112.7	3.4	24.3	57.7	-22.6	Blackbird	*QUt*.*spa-6B*.*2*
T33	2011	6B	Tdurum_contig76997_244	110.7	3.4	25.6	64.8	-15.2	Blackbird	*QUt*.*spa-6B*.*2*
T33	2012	6B	Tdurum_contig76997_244—barc24	112.7	3.6	23.5	56.6	-20.5	Blackbird	*QUt*.*spa-6B*.*2*
Mixture H1^f^	2011	6B	Tdurum_contig76997_244—barc24	112.7	3.5	29.2	74.9	-22.7	Blackbird	*QUt*.*spa-6B*.*2*
Mixture H2	2011	6B	Tdurum_contig76997_244—barc24	112.7	3.5	18.2	59.3	-21.0	Blackbird	*QUt*.*spa-6B*.*2*
Mixture H1	2012	6B	Tdurum_contig76997_244—barc24	114.7	3.4	21.9	61.0	-22.0	Blackbird	*QUt*.*spa-6B*.*2*
Mixture H2	2012	6B	Tdurum_contig76997_244	110.7	3.4	17.3	55.1	-21.4	Blackbird	*QUt*.*spa-6B*.*2*
T33	2012	7A	Tdurum_contig67992_182	66.6	3.6	6.0	8.0	-7.8	Blackbird	*QUt*.*spa-7A*.*2*

^a^ nearest marker to the QTL position is underlined

^b^ based on 1,000 permutations

^c^ maximum likelihood LOD score for the QTL

^d^ phenotypic variation explained by the QTL

^e^ positive values indicate Strongfield allele, and conversely, negative values indicate Blackbird allele that minimize the loose smut incidence of corresponding *Ustilago tritici* race(s)

^f^ H1 and H2 are two heads inoculated at the same time but the seed was maintained and grown separately for assessment

**Fig 3:**
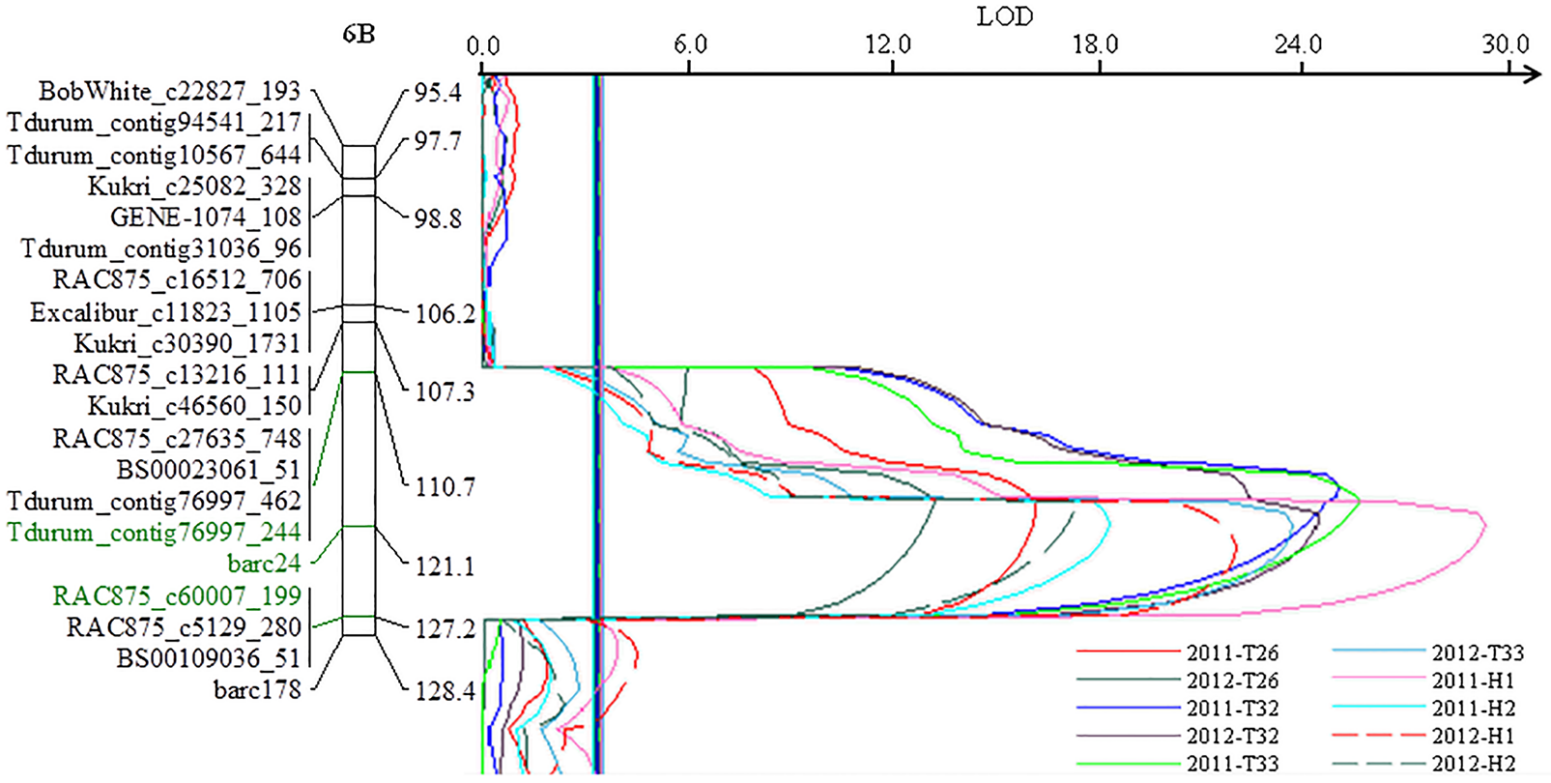
A QTL for loose smut resistance detected on chromosome 6B. A region of the chromosome 6B genetic map showing the position of SNP and SSR markers and highly significant QTL (*QUt*.*spa-6B*.*2*) associated with resistance to multiple races (T26, T32 and T33) of *Ustilago tritici*. Markers associated with the QTL are shown in green to the left of the double line representing the chromosome and their positions along the map are given to the right. A thick and multi-colored vertical line below the QTL peaks represents the genome-wide LOD threshold values (range 3.28–3.55) for declaring significant QTL.

A major and stable QTL, *QUt*.*spa-6B*.*2*, was located in interval *RAC875_c13216_111*—*barc24* (110.7 cM) on chromosome 6B for resistance to all three *U*. *tritici* races: T26, T32 and T33 (Fig 3). The parent ‘Blackbird’ contributed the resistance of *QUt*.*spa-6B*.*2* and this QTL allele accounted for a maximum reduction of loose smut incidence of 74.9% in the partial vacuum inoculation of mixed races in 2011. The QTL, *QUt*.*spa-6B*.*2*, produced the greatest reduction in loose smut incidence and the effect was consistent for different races and types of inoculation over the two environments. Individual race PV explained by *QUt*.*spa-6B*.*2* with race T26 was lower (35–39.2%) compared to races T32 and T33 (56.6–64.8%).

A QTL, *QUt*.*spa-3A*.*2*, derived from ‘Strongfield’ for resistance to race T26 in each trial was detected on chromosome 3A at markers *Kukri_c10751_264* (150.5 cM) and *JG_c2645_107* (151.9 cM). This QTL explained up to 21% of the phenotypic variation of *U*. *tritici* race T26, which was lower than the ‘Blackbird’ QTL mapped on chromosome 6B with the same race. A minor QTL designated as *QUt*.*spa-7A*.*2* mapped on chromosome 7A was identified for resistance to race T33 and associated with marker *Tdurum_contig67992_182* positioned at 66.6 cM. The resistant allele of the QTL on chromosome 7A was contributed by the resistant parent ‘Blackbird’.

## Discussion

The high level of disease in the susceptible checks and DH lines of the Strongfield/Blackbird population for individual race and mixed race inoculations demonstrated the suspensions of *U*. *tritici* spores were viable and optimum conditions were met for LSI in field environments in both 2011 and 2012. The complete resistance phenotype of ‘Blackbird’ to loose smut races T32 and T33 and near complete resistance to race T26 indicated Blackbird possesses a very effective form of resistance. The effectiveness against all three races suggests a broad form of resistance.

The common segregation of the strong QTL from ‘Blackbird’ to races T26, T32 and T33 suggests a single major genetic factor with broad resistance within the 6B locus (Fig 3). Resistance to the mixture of races demonstrates the resistance will stand up against virulence recombinants that would have occurred with multiple races being present in the same floret. Broad, large effect major genes have been reported previously and are included in a review by Knox and Menzies [9]. The 6B QTL is in the same region as a QTL identified by Knox et al. [16] spanning *barc24*. The QTL identified as *QUt*.*spa-6B* derived from the genotype SC8021-V2 [49] and was effective to *U*. *tritici* race T39 [16]. Consequently, we have assigned the label *QUt*.*spa-6B*.*2* to the 6B Blackbird locus to distinguish the two QTL. *QUt*.*spa-6B*.*2* appears to be stable with consistently strong expression in each environment against all three races and their mixture. Knox et al. [16] named the gene responsible for *QUt*.*spa-6B* as *Ut9*. Although the similar location of *QUt*.*spa-6B* and *QUt*.*spa-6B*.*2* suggests the same gene, further work is required to confirm this hypothesis.

In addition to *QUt*.*spa-6B*.*2*, other resistance QTL were observed which is consistent with the pattern of phenotypic segregation. Although Strongfield was susceptible to races T32 and T33, it showed incomplete resistance to race T26. The transgressive segregation between ‘Strongfield’ and ‘Blackbird’ indicated a difference in the resistance against race T26. The analysis confirmed this observation, with QTL derived from each parent identified on different chromosomes indicating more than one gene segregating in the population controlling the incidence of T26. Whereas the resistance in Blackbird, *QUt*.*spa-6B*.*2*, was associated with chromosome 6B, the resistance in Strongfield, *QUt*.*spa-3A*.*2*, was associated with chromosome 3A. Knox et al. [16] identified a QTL named *QUt*.*spa-3A* for resistance to three races T9, T19 and T39 of *U*. *tritici* from the cultivar Glenlea. To distinguish the QTL contributed by Strongfield from that of Glenlea, we designate the Strongfield QTL *QUt*.*spa-3A*.*2*. Although stable in this study, unlike *QUt*.*spa-6B*.*2*, *QUt*.*spa-3A*.*2* was characterized as narrow resistance with effectiveness against only one of the three races. Knox et al. [16] designated the gene within QTL *QUt*.*spa-3A* as *Ut8* and although it appears in the same region as *QUt*.*spa-3A*.*2* close to marker *wmc559* further work is required to determine if *QUt*.*spa-3A* and *QUt*.*spa-3A*.*2* are the result of the expression of the same gene.

The third QTL, *QUt*.*spa-7A*.*2*, which came from ‘Blackbird’ and detected on chromosome 7A, was not as stable as the 6B and 3A resistance being that it was only detected in 2012. *QUt*.*spa-7A*.*2* expressed narrow resistance, effective on race T33 only. Dhitaphichit et al. [50] reported resistance to *U*. *tritici* on chromosome 7A in Hope by using Hope/Chinese Spring substitution lines. Knox et al. [16] identified a major QTL on chromosome 7A named *QUt*.*spa-7A* for resistance to race T9 of *U*. *tritici* and designated the gene within as *Ut7* derived from the genotype SC8021-V2 [49]. To distinguish the two QTL, we designate the Blackbird QTL *QUt*.*spa-7A*.*2*. As SC8021-V2, Glenlea, 9340-CP* and Hope are all hexaploid wheat, understanding the relationship between the hexaploid and tetraploid wheat genes for loose smut resistance requires further research.

Gene-derived marker assays, such as the 90K Infinium iSelect [35], offer an opportunity to construct a transcript SNP map and to explore genome-wide trait variation in complex genomes. The probes arrayed on the Infinium chip are derived from gene transcripts of hexaploid and tetraploid wheat, and *Aegilops tauschii*. Of the total 81,587 SNPs, Wang et al. [35] attempted *in silico* mapping and assigned 55,038 SNP markers to the A- and B-genomes, which corresponds to the genomes of durum wheat. Zanke et al. [51] in wheat and Lin et al. [52] in oat (*Avena sativa*) demonstrated the successful application of a 90K SNP assay in mapping and QTL identification. High-throughput SNP genotyping using the 90K Infinium wheat assay also was successfully employed in the present study. Using a version of GenomeStudio software for diploids, genotyping for 86.5% of the features was achieved on 92 DNA samples. This success rate was comparable with other reports using low to high-density Infinium SNP genotyping arrays conducted in several plant species including wheat [34, 35, 39], maize [53], walnut [54], cherry [55], apple [56]; peach [57]. From a total of 81,587 assays on the Infinium array, 84.2% were categorized as monomorphic, heterozygous, poor call and failed, while the remaining 15.8% were found to be polymorphic. Based on the 90K assay, the number of polymorphic SNPs found in the present study was in good agreement with Russo et al. [58] using a mapping population of 136 recombinant inbred lines. A possible reason for a large number of SNP failures is the absence of probe binding sites in template DNA. For example, SNP probes specific to *T*. *aestivum* and *Ae*. *tauschii* genomes are less likely to anneal in tetraploid wheat thus giving a failed or null call. The polymorphic SNPs detected in this study of tetraploid wheat were high-quality and included: (i) simple SNPs representing straightforward allelic variation in the parents and progenies, (ii) hemi-SNPs characterized by one of two of the homoeologous chromosomes harbouring a SNP, and (iii) those showing the presence and absence polymorphism with a segregation ratio of 1:1.

The linear order and assignment of markers of SNP and SSR markers were in general agreement with maps published based on the 90K assay for tetraploid [42, 58, 59] and hexaploid [35, 39] wheat. The complete genetic map of SNP/SSR markers spanning 3008.4 cM with an average genetic distance of 0.2 cM between markers reduces the gaps between markers compared to previously published durum maps [42, 60–63]. However, there are some genetic regions where large gaps still exist in the present map. For instance, the two unconnected linkage groups for each of the 3B and 7A chromosomes indicated the existence of a large gap. The gaps may be attributed to either these regions not being adequately represented in the 90K assay or as a result of lack of polymorphism between Strongfield and Blackbird.

As indicated in the earlier studies, the total number of markers on the map of the B-genome (4945) was higher than the map of the A-genome (3969) of wheat [42, 64]. Both genomes were nearly similar in total genetic length with 1696.3 cM for the A-genome and 1681.3 cM for the B-genome, which is consistent with findings of Maccaferri et al. [42].

High-density molecular maps improving precision in QTL detection has been recently demonstrated in several crops [52, 65–69]. Ours is the first attempt in durum wheat to identify and map loose smut QTL using a high-density SNP/SSR genetic map. Since 411 SSR markers were integrated in the SNP map, the cross links between SNP and SSR markers can be used for comparative mapping of QTL linked to SSR markers reported in earlier studies.

In this study, we set out to use a high-density SNP array to generate markers to map loose smut resistance segregating in a population derived from Strongfield/Blackbird. The results not only revealed three loci contributing to loose smut resistance in tetraploid wheat, but confirm the value of a high-density map based on SNP markers. We presented the successful application of array-based SNP genotyping to the identification and mapping of a major QTL (*QUt*.*spa-6B*.*2*) on chromosome 6B with broad resistance in *T*. *turgidum* L. subsp. *carthlicum* wheat to loose smut races T26, T32 and T33. The gene and markers associated with this QTL have potential for use in marker-assisted selection and genomic predictions in durum wheat breeding programs for resistance to *U*. *tritici*. Additional effort will be necessary to convert the co-localized SNPs into breeder-friendly functional markers for screening genotypes with loose smut resistance. Further, the results of this study will contribute to the eventual understanding of the relationship of the Blackbird QTL with the gene *Ut9* on chromosome 6B.

## Supporting information

S1 Fig Infinium II iSelect 90K SNP assay clustering patterns. Representative examples from the observed cluster patterns based on GenomeStudio in Strongfield/Blackbird DH population. The positions of genotype clusters are indicated by eclipse and clusters of red dots are AA calls, blue dots are BB calls and AB calls are shown by purple dots. **(a)** A SNP, Bobwhite_c34661_208, showing an example pattern with separation of two distinct clusters AA and BB alleles. **(b)** A SNP, Bobwhite_c17191_297, showing an example pattern with separation of two distinct clusters of AA and AB. **(c)** A SNP, RAC875_c9790_116, showing an example of presence and absence polymorphism of AA and null allele. In each graph a, b and c, the progeny segregation agrees with an expected 1:1 ratio. **(d)** SNP graph showed a failed SNP, BobWhite_rep_c63688_365, that could not anneal with the target DNA region and therefore no fluorescent signal was captured. Parents ‘Strongfield’ and ‘Blackbird’ are indicated by lime and magenta colors, respectively.
(TIF)

S2 Fig Genetic linkage map. Distribution of 12,445 markers (12,034 SNP and 411 SSRs) on 16 linkage groups that represent 14 chromosomes of the Strongfield/Blackbird DH population. Horizontal lines within the chromosome bar correspond to individual markers. Bold horizontal lines indicate a group of co-segregating markers (multiple markers mapped to the same place). Genetic distance (cM) is shown on the left of each chromosome bar.
(TIF)

S1 Table The mean percent loose smut incidence (LSI, %) of loose smut for parents and check cultivars for each race and race combination. (DOCX)

S2 Table Complete Genetic linkage map of SNP and SSR markers and their positions (cM) for the 14 Strongfield/Blackbird chromosomes. (XLSX)
