# Supplementary figures and images for: High-density genetic mapping of a major QTL for resistance to multiple races of loose smut in a tetraploid wheat cross
Source: PLoS One. 2018 Feb 27;13(2):e0192261. doi: 10.1371/journal.pone.0192261 (PMC5828438; doi:10.1371/journal.pone.0192261)

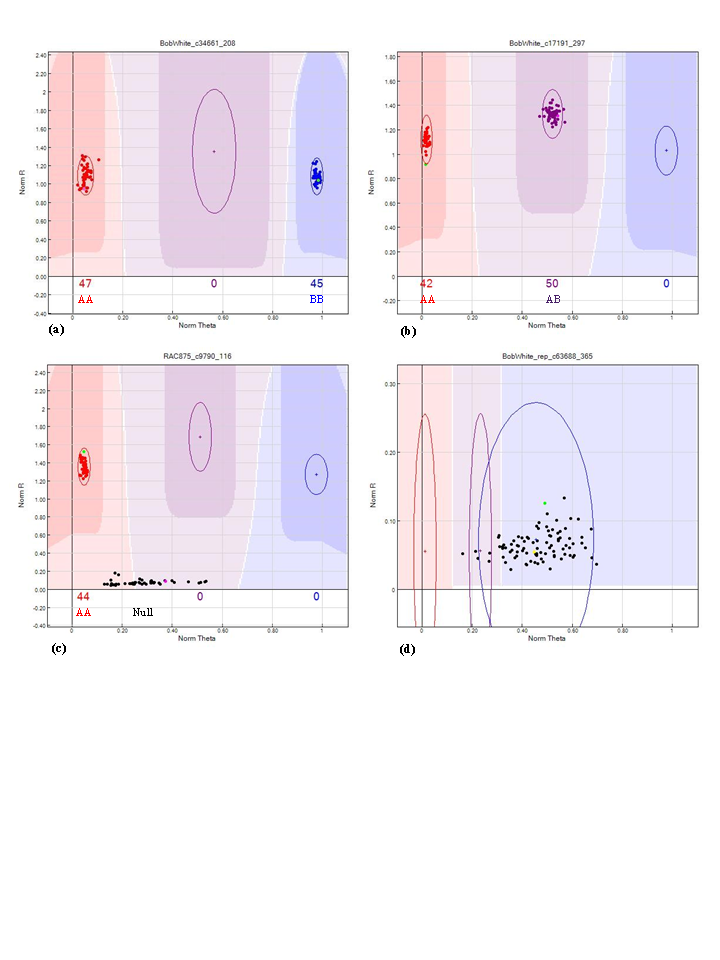

Supplement: S1 Fig — Representative examples from the observed cluster patterns based on GenomeStudio in Strongfield/Blackbird DH population. The positions of genotype clusters are indicated by eclipse and clusters of red dots are AA calls, blue dots are BB calls and AB calls are shown by purple dots. (a) A SNP, Bobwhite_c34661_208, showing an example pattern with separation of two distinct clusters AA and BB alleles. (b) A SNP, Bobwhite_c17191_297, showing an example pattern with separation of two distinct clusters of AA and AB. (c) A SNP, RAC875_c9790_116, showing an example of presence and absence polymorphism of AA and null allele. In each graph a, b and c, the progeny segregation agrees with an expected 1:1 ratio. (d) SNP graph showed a failed SNP, BobWhite_rep_c63688_365, that could not anneal with the target DNA region and therefore no fluorescent signal was captured. Parents ‘Strongfield’ and ‘Blackbird’ are indicated by lime and magenta colors, respectively. (TIF) [file pone.0192261.s001.TIF]

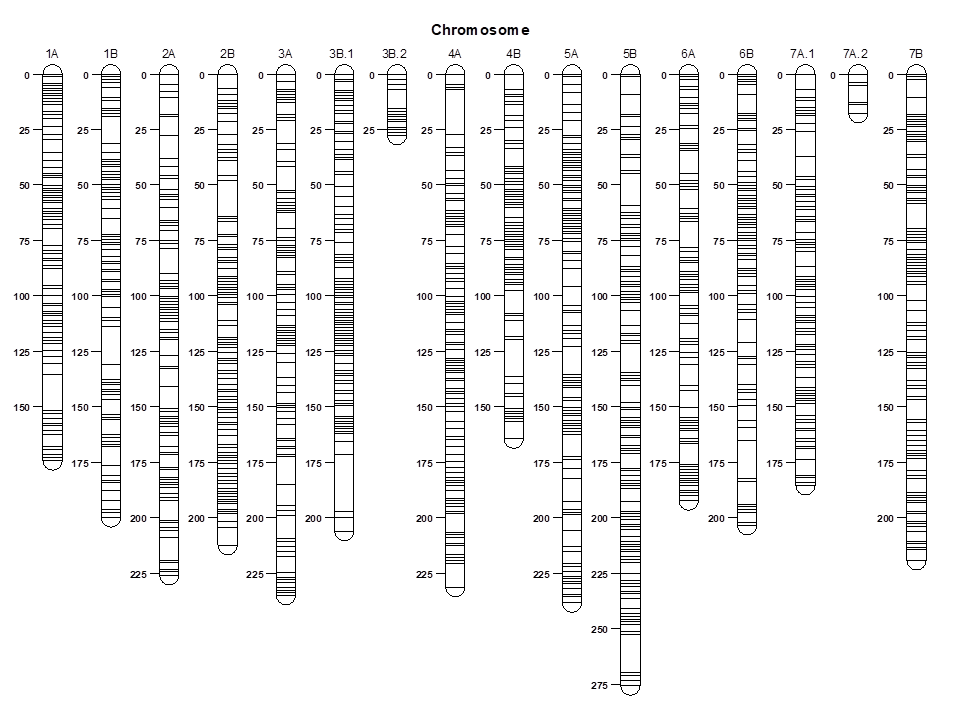

Supplement: S2 Fig — Distribution of 12,445 markers (12,034 SNP and 411 SSRs) on 16 linkage groups that represent 14 chromosomes of the Strongfield/Blackbird DH population. Horizontal lines within the chromosome bar correspond to individual markers. Bold horizontal lines indicate a group of co-segregating markers (multiple markers mapped to the same place). Genetic distance (cM) is shown on the left of each chromosome bar. (TIF) [file pone.0192261.s002.TIF]
