# Supplementary material for: High-density genetic mapping of a major QTL for resistance to multiple races of loose smut in a tetraploid wheat cross
Source: PLoS One. 2018 Feb 27;13(2):e0192261. doi: 10.1371/journal.pone.0192261 (PMC5828438; doi:10.1371/journal.pone.0192261)
Supplement: S1 Table — (DOCX) [file pone.0192261.s003.docx]

**Table S1.** The mean percent incidence (%) of loose smut for parents and check cultivars for each race and race combination.

| Parent and check cultivars | Syringe inoculation | | | | | | | |  | Vacuum inoculation^*^ | | | | |
| --- | --- | --- | --- | --- | --- | --- | --- | --- | --- | --- | --- | --- | --- | --- |
|  | T26^a^ | |  | T32 | |  | T33 | |  | H1^b^ | |  | H2 | |
|  | 2011 | 2012 |  | 2011 | 2012 |  | 2011 | 2012 |  | 2011 | 2012 |  | 2011 | 2012 |
| Blackbird | 5 | 2 |  | 0 | 0 |  | 6 | 0 |  | 2 | 0 |  | 0 | 0 |
| Strongfield | 6 | 9 |  | 77 | 35 |  | 41 | 92 |  | 46 | 42 |  | 83 | 13 |
| Brigade | 19 | 14 |  | 73 | 65 |  | 44 | 71 |  | 83 | 67 |  | 50 | 61 |
| Commander | 11 | 6 |  | 65 | 83 |  | 43 | 36 |  | 25 | 41 |  | 37 | 29 |
| DT696 | 9 | 3 |  | 63 | 89 |  | 63 | 61 |  | 57 | 45 |  | 80 | 42 |
| AAC Raymore | 8 | 1 |  | 89 | 20 |  | 49 | 54 |  | 46 | 52 |  | 61 | 53 |

^*^Vacuum inoculation refers mixture of races T26, T32 and T33

^a^ Loose smut race designation

^b^ H1 = head 1; H2 = head 2
